# Validation of the Flexible and Rigid Cognitive Restraint Scales in a General French Population

**DOI:** 10.3390/ijerph191912519

**Published:** 2022-09-30

**Authors:** Sandrine Péneau, Marc Bénard, Margaux Robert, Benjamin Allès, Valentina A. Andreeva, Frédéric Courtois, Mathilde Touvier, Christophe Leys, France Bellisle

**Affiliations:** 1 Sorbonne Paris Nord University, INSERM U1153, INRAE U1125, CNAM, Nutritional Epidemiology Research Team (EREN), Epidemiology and Statistics Research Center—University of Paris (CRESS), 93017 Bobigny, France; 2 Faculty of Psychological Sciences, Université Libre de Bruxelles, Avenue Franklin Roosevelt, 50-CP191, 1050 Brussels, Belgium

**Keywords:** dietary restraint, Three-Factor Eating Questionnaire, eating behavior, psychometrics, validation

## Abstract

Distinguishing between flexible and rigid cognitive restraint (CR) may be useful for understanding the role of CR in dietary behavior and weight status. This study aimed to translate and adapt the flexible and rigid CR scales to the French context and test their psychometric properties. Construct validity, internal consistency, and test–retest reliability were examined in a sample of 620 individuals. Confirmatory factor analysis of the scales found a two-factor structure (flexible CR: 12 items; rigid CR: 15 items) that provided a good fit and supported the initial solution (*χ*^2^ = 584.7, df = 322, CFI = 0.96, RMSEA = 0.052 [0.045, 0.059], TLI = 0.95). Higher flexible and rigid CR were associated with higher CR overall, emotional eating (TFEQ-R21) and eating disorders (SCOFF), and lower intuitive eating (IES-2). In addition, higher flexible CR was associated with lower impulsivity (BIS-11) while higher rigid CR was associated with higher uncontrolled eating (TFEQ-R21) and lower self-esteem (RSES), satisfaction with life (SWLS), and optimism (LOT-R). Flexible and rigid CR internal consistency was satisfactory (McDonald ω = 0.77 and 0.74, respectively) and test–retest reliability was good (ICC = 0.81 and 0.79, respectively). This study validated a flexible and rigid CR tool in a French population and confirmed that these two types of CR represent distinct eating behaviors.

## 1. Introduction

Many individuals display a chronic tendency to limit food intake in order to control their body weight. The social context where thinness is perceived as an ideal might be one of the predisposing factors leading to this behavior. The term “restrained eating” was first introduced in the mid-1970s by Herman and Mack to describe this tendency [1]. Deliberate and chronic restriction of food intake has been suggested to induce counter-regulatory responses, reduce sensitivity to satiety signals and result in disinhibited, binge-like eating patterns. [2]. For decades, an abundant literature has argued that dietary restraint is a major cause of weight and psychological problems. However, cross-sectional studies have been contradictory and thus far have shown positive [3,4,5], negative [6], or null associations [4] between cognitive restraint (CR) and weight status. It has been suggested that CR could constitute a risk factor [3,7] or a consequence of weight gain [4,8], while some evidence suggests that these two constructs are not associated [4,6,9,10,11]. Associations have consistently been shown to vary between obese and normal-weight populations [4,5,12,13].

The potentially deleterious effect of restraint has been attributed to the co-occurrence of restriction and disinhibition, the latter describing the occasional hyperphagia that occurs as a result of a breakdown of inhibition in individuals who diet rigidly and then suddenly lose control and overeat in response to different types of stimuli [14,15]. The conflicting evidence in the literature has led researchers to break down the restrained eating construct in order to identify features associated with positive and negative outcomes. Westenhoefer et al. developed two scales based upon the extent of their correlation with the disinhibition factor [16,17]: a rigid control scale exhibiting a stronger correlation with disinhibition compared with a flexible control scale. Rigid control is characterized by a dichotomous, all-or-nothing approach to eating, dieting, and weight status. Flexible control, on the other hand, is characterized by a more graduated, permissive approach where “fattening” foods are eaten in limited quantities without feelings of guilt.

Subsequent studies have confirmed that rigid CR was positively associated with disinhibition, whereas flexible CR typically showed a negative or null association [18,19]. In addition, rigid CR was associated with higher weight status [12,19,20,21,22,23], while flexible CR was associated with lower weight status [12,19,21,23,24] or showed no association [12,20,22]. However, these findings are somewhat inconsistent, as both types of CR have been associated negatively [6,25,26] or positively [18] with weight in different study samples. In addition, flexible CR was associated with greater weight loss compared with rigid CR [23]. It must be noted that many CR studies used an early version of the scales which demonstrated modest internal consistency and high correlations among rigid and flexible CR [12,20,21]. In addition, those studies were based on small and/or very specific samples such as women, adolescents or individuals with eating disorders [18,20,21,23,25]. Hence, authors have called for more research in this field in diverse populations [27,28], given evidence that the distinction of flexible and rigid control may be useful in better understanding the role of restraint in dietary behavior and weight management. In order to accurately measure these constructs in a French-speaking population, the questionnaire ought to be cross-culturally adapted with further evaluation of the validity of the translated instrument [29].

The aims of the present study were therefore to adapt the flexible and the rigid CR scales to the French context and examine their psychometric properties in a sample derived from the general population. Specifically, we evaluated the construct validity of the translated instrument, i.e., studying its factor structure, comparing scores between subgroups with “a priori” differences in flexible and rigid CR, and testing its correlation with other scales assessing eating behaviors and psychological well-being. We also examined the instrument’s internal consistency and test–retest reliability.

## 2. Methods

### 2.1. Population

This study was conducted within the NutriNet-Santé study, which is a large, ongoing web-based prospective cohort started in France in May 2009. The rationale, design and methods of the study have been described elsewhere [30]. Its overall aim is to explore the relationships between nutrition and health and the determinants of eating behavior and nutritional status. Participants are adult volunteers (age ≥ 18 years) recruited from the general French population. At inclusion, they are asked to complete a set of self-reported web-based questionnaires to assess their diet, health status, physical activity, anthropometric data, socio-economic conditions, and lifestyle characteristics. In addition, optional questionnaires related to eating behavior determinants and specific health-related outcomes are sent each month. In this context, the translated CR questionnaire was sent to a random subsample of 1000 NutriNet-Santé participants in January 2017. This subsample was representative of the French population in terms of age, gender, and level of education [31]. The CR questionnaire was sent a second time (one and a half month later) to the sub-sample of individuals who had completed the questionnaire. All other measures, i.e., socio-demographic, anthropometric, eating behaviors, and psychological well-being variables, were collected among the whole cohort between 2010 and 2017. The NutriNet-Santé study is conducted in accordance with the guidelines of the Declaration of Helsinki, and all study procedures were approved by the Institutional Review Board of the French Institute for Health and Medical Research (IRB Inserm n° 0000388FWA00005831) and by the Commission Nationale Informatique et Libertés (CNIL n° 908450 and n° 909216). Electronic informed consent was obtained from all participants. The NutriNet-Santé study is registered in ClinicalTrials (N°NCT03335644).

### 2.2. Instruments

#### 2.2.1. Flexible and Rigid CR Scales

##### Questionnaire Items

The original flexible and rigid CR scales were developed and validated by Westenhoefer et al. [17] as an extension of the CR dimension of the Three-Factor Eating Questionnaire (Eating Inventory) [32]. Flexible CR is “characterized by a graduated approach to eating, dieting, and weight, in which ‘fattening’ foods are eaten in limited quantities without feelings of guilt,” while rigid CR is “characterized by a dichotomous, all-or-nothing approach to eating, dieting, and weight” [17]. The flexible and rigid CR scales comprise 12 and 16 items, respectively. Overall, 18 items are rated on a 2-point scale (true/false) while 10 items are rated on a 4-point scale using various anchor words (see Supplemental Tables S1 and S2). All items are dichotomously coded for analysis. The total score is obtained by first summing up all individual item ratings and then dividing the result by the total number of items in each scale, for a final score ranging from 0 to 12 for the flexible CR, and from 0 to 16 for the rigid CR (higher scores indicating greater control).

##### French Adaptation Protocol

The flexible and rigid CR dimensions were cross-culturally adapted from English to French following the guidelines by Beaton et al. [29]. Forward translations were independently performed by two bilingual translators informed about the concepts underlying the questionnaire, and by one bilingual translator naïve to the concepts being measured; all three translators were native French speakers, specializing in nutrition. A synthesis of the three translations was created. Then, two bilingual native English translators, unfamiliar with the original English version, backtranslated the synthesized French questionnaire. All translations were reviewed by an expert committee composed of all translators in order to develop the pre-final version of the questionnaire. Dr. Joachim Westenhoefer, who developed and validated the original scales [17], provided feedback and advice during the scale adaptation process and gave the permission to modify it. Next, the questionnaire was pre-tested by 11 French-speaking community members in order to evaluate item comprehension. Overall, all items and response modalities were well understood. A few changes in item formulations, in accordance with French language use conventions, were performed, as follows: “my quota of calories” was changed to “the quantity of calories I need” (FC1), “light food” was changed to “low-calorie food” (FC10), “without a diet plan” was changed to “without a diet” (RC15). Response modalities were also slightly modified: a modality was added for participants to whom the situation described by the item did not apply (e.g., “I have never followed a diet”) (FC3, RC5, RC13, RC15), some modalities were presented in reverse order (FC12), and some modalities were slightly changed in order to better fit the question or cover a wider range of behaviors (FC6, RC3, RC4, RC6, RC7).

#### 2.2.2. Other Variables

##### Cognitive Restraint, Emotional Eating, Uncontrolled Eating

Participants completed the French version of the revised 21-item Three-Factor Eating Questionnaire (TFEQ-R21) [33] approximately 14 months after inclusion in NutriNet-Santé (between July 2010 and January 2011 for most participants). This questionnaire covers the following 3 aspects of eating behavior: overall CR (6 items), emotional eating (6 items), and uncontrolled eating (9 items). The items are assessed on 4-point scales that range from definitely true to definitely false. Raw scores are transformed to a 0–100 scale ([raw score-min score]/score range × 100). Higher scores indicate greater CR, emotional eating, and uncontrolled eating. In our sample, McDonald ω coefficients (along with associated 95% confidence intervals, CI) showed good internal consistency for each scale: CR: ω = 0.79 (%95 CI: 0.76, 0.81); emotional eating: ω = 0.94 (%95 CI: 0.92, 0.94), and uncontrolled eating: ω = 0.84 (%95CI: 0.82, 0.86).

##### Intuitive Eating

Intuitive eating is defined as generally eating in response to physiological hunger and satiety signals rather than external and/or emotional cues, along with giving oneself unconditional permission to eat desired food when hungry [34,35]. It was assessed by the validated French version [36] of the Intuitive Eating Scale-2 (IES-2) [37]. Participants completed the questionnaire between December 2013 and June 2014. The French IES-2 includes 3 dimensions: eating for physical rather than emotional reasons (8 items), reliance on hunger and satiety cues (6 items), and unconditional permission to eat (4 items). Items are rated on a 5-point Likert scale ranging from “strongly disagree” to “strongly agree.” IES-2 total score as well as subscale scores range from 1 (low intuitive eating) to 5 (high intuitive eating). The scale showed good internal consistency: IES-2 total score: ω = 0.83, 95% CI: 0.80, 0.86, eating for physical rather than emotional reasons: ω = 0.91 (95% CI: 0.89, 0.93), reliance on hunger and satiety cues ω = 0.86 (95% CI: 0.84, 0.88), and unconditional permission to eat ω = 0.61 (95% CI: 0.55, 0.67).

##### Impulsivity

Impulsivity was assessed with the French version [38] of the Barratt Impulsiveness Scale (BIS-11) [39], derived from the French version of the BIS-10 questionnaire. Participants completed the questionnaire between June and December 2014. It is a 30-item self-report questionnaire developed to assess the personality-based construct of impulsivity. Each item is scored on a 4-point Likert scale ranging from “rarely/never” to “almost always/always.” The final score ranges from 1 to 4, with higher scores indicating greater impulsivity. The scale displayed good internal consistency (ω = 0.78, 95% CI: 0.75, 0.81).

##### Eating Disorders

The French version [40] of the SCOFF questionnaire [41] was completed by participants between April and October 2017. It is a self-report questionnaire with five dichotomous (Yes/No) questions designed to screen for risk of eating disorders. The total score ranges from 0 to 5, where a score equal to or greater than 2 indicates risk of eating disorders.

##### Self-Esteem

The French version [42] of the Rosenberg Self-Esteem Scale (RSES) [43] was completed by participants between October and December 2016. It features one global dimension of self-esteem evaluated with 10 items rated on a 4-point Likert scale ranging from “strongly disagree” to “strongly agree.” The final score ranges from 10 (poor self-esteem) to 40 (excellent self-esteem). The scale showed good internal consistency (ω = 0.89, 95% CI: 0.87, 0.90).

##### Satisfaction with Life Scale

Participants completed the French version [44] of the Satisfaction with Life Scale (SWLS) [45] between October and December 2016. It provides a global assessment of a person’s quality of life according to his/her chosen criteria. The questionnaire consists of 5 items rated on a 7-point Likert scale ranging from “strongly agree” to “strongly disagree.” The total score ranges from 5 (very low satisfaction with life) to 35 (very high satisfaction with life). The scale’s internal consistency was very good (ω = 0.90, 95% CI: 0.87, 0.91)).

##### Optimism

The French version [46] of the Life Orientation Test-Revised (LOT-R) [47] was completed between October and December 2016. The questionnaire assesses dispositional optimism, which can be defined as a general expectation that good things will happen in the future [48]. The LOT-R consists of 3 items measuring optimism, and 3 items measuring pessimism, all rated on a 5-point Likert scale. Response options range from “strongly agree” to “strongly disagree”. The score for each subscale is obtained by adding the individual scores of the respective items. After reverse-coding the pessimism score, a total score is calculated. The LOT-R had good internal consistency (ω = 0.84, 95% CI: 0.82, 0.87).

##### Socio-Demographic, Anthropometric, and Lifestyle Data

Socio-demographic, anthropometric and lifestyle data were self-reported each year, using online tools that were pilot-tested and validated against traditional assessment methods [49,50] and against clinical measurements for the anthropometric data [51]. The latest available socio-demographic/anthropometric/lifestyle data to the date of completion of the flexible and rigid CR scales were used. Collected information included: age (years), gender, educational level (primary, secondary, undergraduate, and postgraduate), occupational status (unemployed, student, self-employed or farmer, employee and manual worker, intermediate profession, managerial staff or intellectual profession, and retired), weight, height, and history of dieting to lose weight (never/ever). Body mass index (BMI, kg/m^2^) was calculated as the ratio of the self-reported weight in kg to the squared height in m.

### 2.3. Statistical Analysis

#### 2.3.1. Construct Validity

##### Structural Validity

A CFA seldom yield a robust model in the first try [52]. Indeed, it is normal to have some correlated error terms between items of a given factor. However, it is impossible to predict which correlation between error terms should be estimated prior to a first CFA analysis. After this first try, one should consider the modification indices and detect which correlations must be estimated to yield a better fit. Therefore, this step puts us back in an exploratory logic. To conduct a fully confirmatory analysis, we split our sample into two random sub-samples (given that we had a large sample available). In order to test the initial two-factor model (rigid versus flexible CR), and considering the non-normality of the value distribution, we performed a first confirmatory factor analysis (CFA) on one of the sub-samples using the mean- and variance-weight least square estimator (WLSMV). It is equivalent to the diagonally weighted least-square estimator with robust standard errors and mean- and variance-adjusted test statistics. As applied to graded response models, this method uses the tetrachoric correlation matrix and is better suited for categorical variables compared with the maximum likelihood estimator which has been shown to bias model-data fit [53]. It is assumed that there is a continuous latent response variable underlying each item. Thresholds are calculated to denote the cut-point (expected value) of these continuous latent variables, which determines a person’s position vis-à-vis each item. Factor variances were fixed at 1.0 and the covariance parameter between factors was free. Goodness- and badness-of-fit indices included the chi-square by degrees of freedom index (*χ*^2^/df), the comparative fit index (CFI), the Tucker-Lewis index (TLI), and the root mean square error of approximation (RMSEA). The standardized root mean square residual (SRMR) was not reported because this index was found to perform poorly with binary outcomes [54]. Estimations were based on the following cutoffs: *χ*^2^/df < 5; CFI > 0.95; TLI > 0.95; and RMSEA < 0.06.

Inter-item tetrachoric correlations were calculated to identify items with too weak (r < 0.30) (suggesting lack of association) or too strong (r > 0.80) correlations (suggesting redundancy), leading to the suppression of one of these items. Furthermore, to identify potential sources of misspecification, standardized coefficients (standardized regression slopes), standardized residuals (standardized difference between observed and model-implied variance for each item), modification indices (approximations of the extent to which the overall model *χ*^2^ would decrease if fixed parameters were freely estimated), expected parameter change values (approximations of expected coefficient value if fixed parameters were freely estimated), and theoretical considerations were explored [53].

Once we conducted this first “exploratory CFA” and adjusted the model we performed a new CFA on the second subsample. This step, with the irrelevant items removed and all relevant correlations between error terms estimated, allowed us to test the CFA on a new set of data, thereby supporting a fully confirmatory analysis.

##### Hypothesis Testing

Differences in total scores on each scale according to socio-demographic (gender, age, educational level, occupational status) and anthropometric characteristics (BMI) were evaluated with ANOVA to estimate convergent and discriminant validity. Convergent and discriminant validity was further assessed by calculating Pearson’s correlations, with the original subscales of the TFEQ-R21 assessing CR, emotional eating and uncontrolled eating, the IES-2 (overall and with each subscale) measuring intuitive eating, the BIS-11 measuring impulsivity, the SCOFF measuring eating disorders, the RSES measuring self-esteem, the SWLS measuring satisfaction with life and the LOT-R measuring optimism. Since interactions between gender and each scale were not significant, analyses were not stratified.

#### 2.3.2. Reliability

Scale reliability was assessed with the McDonald’s ω [55]. Test-retest data were collected from the participants who completed the questionnaire twice. The level of consistency between the successive measurements was estimated with the intraclass correlation coefficient (ICC), calculated as the ratio of the subject variance by the sum of the subject variance and the residual variance.

#### 2.3.3. Software

Hypotheses were specified before the data were collected and the analytic plan was pre-specified. All tests of statistical significance were 2-sided and the significance level was set at 5%. Statistical analyses were performed using R software (Chambers, Murray Hill, NJ, USA). CFA were performed with the lavaan package for latent variable modelling (version 0.5–22, Rosseel, Ghent, Belgium) [56] and reliability coefficients were calculated with the MBESS package, applicable to the behavioral sciences (version 4.1.0, Kelley, Bloomington, IN, USA) [57].

## 3. Results

### 3.1. Sample Description

Among the original 1000 randomly drawn participants, 620 (296 men and 324 women) completed the CR questionnaire in 2017 (62% of those who received it). Mean age was 52.4 years (SD = 15.2), 19.7% had postgraduate education, 16.9%-undergraduate education, 56.5%-secondary education, and 6.9%-primary education. Mean BMI was 24.9 kg/m^2^ (SD = 4.8). A total of 337 participants (54% of those who received it) completed the questionnaire the second time, with a mean test–retest interval of 45 days (SD = 9, range = 22–69 days).

### 3.2. Structural Validity

The original structure of the flexible and rigid CR scales [17] was tested within the first randomly selected subsample. The two-factor model showed a correct fit (Table 1). However, the standardized coefficient for item RC10 (“I would rather skip a meal than stop eating in the middle of one”) was not significant (*p* = 0.67). In addition, inter-item correlations indicated collinearity between items FC8 (“If I eat a little bit more on one day, I make up for it the next day”) and FC11 (“If I eat a little bit more during one meal, I make up for it at the next meal”) (modification indice = 105.69) suggesting that they measured the same concept. Thus, a respecified model, where item RC10 was dropped and the covariance between items FC8 and FC11 was considered, was tested. Covariances do not modify the use of the scales but allow taking into account common errors in the item residual variances, which could be due to similarity in wording. The respecified two-factor model exhibited a good fit (Table 1). The model was further confirmed in the second subsample (Table 1).

Table 2 shows the CFA results of the final two-factor model. The final version of the two CR scales is presented in Supplemental Tables S1 and S2.

### 3.3. Hypothesis Testing

Table 3 shows flexible and rigid CR total scores according to individual socio-demographic, anthropometric and lifestyle characteristics. Overall, women had higher levels of flexible and rigid CR than did men. Flexible CR was higher among older individuals while no difference according to age was observed for rigid CR. No difference was observed according to educational level or occupational status. Rigid CR was lower in normal-weight individuals than in those with either underweight or overweight/obesity, while no difference according to weight status was observed for flexible CR. Finally, flexible and rigid CR were lower in never dieters compared to former or current dieters. Table 4 shows the respective associations of the flexible and rigid CR scales with other scales assessing eating behaviors and psychological well-being. Flexible and rigid CR were positively associated with CR, emotional eating and eating disorders, but negatively associated with intuitive eating (overall and 2 subscales). These associations were overall stronger in the case of rigid CR compared with flexible CR. In addition, flexible CR was also negatively associated with impulsivity while rigid CR was positively associated with uncontrolled eating and negatively associated with self-esteem, satisfaction with life, and optimism.

### 3.4. Reliability Measures

McDonalds ω values indicated satisfactory internal consistency for the flexible (ω = 0.77 [95% CI: 0.76, 0.79]) and rigid CR scales (ω = 0.74 [95% CI: 0.73, 0.76])

The test-retest over a 45-day period revealed good ICC for the flexible (.81 [95% CI: 0.77, 0.85]) and the rigid (0.79 [95% CI: 0.73, 0.83]) subscales.

## 4. Discussion

The flexible and rigid CR scales were translated and validated in a randomly selected subsample of the NutriNet-Santé cohort. The final model included 27 items: 12 for the flexible CR scale and 15 for the rigid CR scale. The construct validity of the respecified two-factor model was good as seen in CFA, hypotheses testing with a comparison of scores between subgroups with “a priori” differences in flexible and rigid CR, and correlation measures of eating behaviors and psychological well-being. Measures of scale reliability showed satisfactory internal consistency and good test-retest reliability.

### 4.1. Cognitive Restraint Is Not a Homogeneous Construct

The original flexible and rigid CR scales, developed by Westenhoefer [17], were consistently reproduced in the present validation study. Our results confirm that CR is not a homogeneous construct and can be differentiated into two types of eating behavior: flexible and rigid. The psychometric and theoretical analysis of the 28-item original tool led to the exclusion of 1 item that had non-significant loading (R10, “I would rather skip a meal than stop eating in the middle of one”).

### 4.2. Scope of Flexible and Rigid CR Scales

According to the initial definition, flexible CR behavior is characterized by a graduated approach to eating, dieting, and weight, in which “fattening” foods are eaten in limited quantities without feelings of guilt [17]. The flexible CR factor was reproduced in the present study. The final 12-item scale assesses tendencies to control weight by limiting the amount of food eaten, selecting low-energy food, compensating from one day to the next, and paying attention to one’s figure. Rigid CR behavior, on the other hand, is characterized by a dichotomous, all-or-nothing approach to eating, dieting, and weight status [17]. The rigid CR factor was reproduced in the present study with the exception of one item. The final 15-item scale assesses a tendency to frequent dieting for weight control purposes, including calorie counting, eating diet food, avoiding stocking up on tempting food, and skipping meals. Item R10 assessing self-control during meals was not included in the final scale. However, the overall meaning of the original rigid CR scale was maintained.

### 4.3. Socio-Demographic Characteristics Are Associated with Flexible and Rigid CR

Bivariate analyses supported prior evidence indicating that women have higher levels of flexible and rigid CR compared with men [6,12,17]. Stronger weight concerns in women compared with men [58] might explain these results. Next, we found no correlation of educational level and occupational status with either flexible or rigid CR, supporting previous data [6]. We found greater flexible CR in older individuals while rigid CR was not associated with age. Mixed results were found in the literature, showing age to be associated with both scales [6], with the rigid scale only [24], or with neither of the two scales [59,60]. The increase of BMI with age [61] might partly account for the greater flexible CR observed in older individuals. Prior results have suggested that rigid CR could be more strongly associated with personality traits, such as anxiety [18] or maturity fears [21], compared with flexible CR. Traits are relatively stable throughout life which could, to some extent, explain the absence of a link between rigid CR and age. The findings also suggest that dietary advice would probably not elicit rigid CR in people who are not predisposed by their personality traits.

### 4.4. Rigid CR Is Associated with Anthropometric Characteristics

Bivariate analyses showed no correlation between flexible CR and weight status. Cross-sectional data in the literature are contrasted, showing null [12,19,20,22], negative [6,12,19,21,24,25] or positive [18] associations with weight status. On the other hand, rigid CR was positively correlated with weight status, in agreement with most (but not all) studies [12,18,19,20,21,22], as some showed a negative [6,25] or null [12,24] association. In previous studies, flexible CR was associated with greater weight loss compared with rigid CR [23], while rigid CR was not associated with weight gain [62]. Finally, we showed higher flexible and rigid CR in dieters, in agreement with the literature [6]. Overall, these cross-sectional results suggest that a flexible approach may be either neutral or beneficial in terms of fostering effective weight control. Flexible restraint may be a self-regulation mediator of weight control, implying the monitoring if one’s food intake [63]. On the other hand, results suggest that engaging in rigid dieting strategies could have a deleterious effect on weight status. Potential mechanisms include increased food cravings and decreased self-regulatory success in weight control with rigid CR [59]. It is unclear however, whether rigid CR is a risk factor or a consequence of weight gain, as debated in the case of overall CR [28].

### 4.5. Flexible and Rigid CR Are Associated with Eating Behavior and Well-Being Characteristics

In the present study, flexible and rigid CR were positively correlated with CR overall, which was expected due to their conceptualization. In addition, both scales were associated with eating disorders, the association with rigid CR being stronger than the one with flexible CR. The literature in this domain mostly focused on bulimia or binge eating, and showed contrasting results with either positive associations for both scales [64] or for the rigid CR scale only [17,19,21,22], or no association [25]. Dichotomous thinking, which characterizes individuals with rigid CR, could contribute to the development of inflexible dietary rules and increase the likelihood of disordered eating following deviations from those rules [65]. Both scales, and the rigid CR scale specifically, were also negatively associated with intuitive eating, supporting previous data [22]. One study, however, showed an association of intuitive eating with the rigid scale only [63], which support our data in the case of “reliance on hunger and satiety cues” dimension. Finally, both scales were positively correlated with emotional eating. Flexible CR was not correlated with other eating behavior or well-being characteristics whereas rigid CR was positively correlated with uncontrolled eating and negatively correlated with self-esteem, satisfaction with life and optimism. This is consistent with previous reports of relationships between rigid CR and eating behavior, eating disorders and psychological traits, whereas flexible CR has shown non-significant results overall [18,21,22,23,25]. In particular, rigid CR was positively associated with disinhibition, a concept close to uncontrolled eating, whereas flexible CR showed a negative or null association [17,18,19]. Other studies have reported associations between greater rigid CR (but not flexible CR) and psychological constructs, such as higher anxiety [18,19], negative affect [22], depressive symptomatology [18,19], preoccupation with body shape [18,23], and lower life satisfaction [22]. We also observed a negative association between flexible CR and impulsivity. In the literature, higher non-planning impulsivity was observed in women with higher CR, while no associations were found for cognitive or motor impulsivity [19]. Overall, these data suggest that rigid CR was associated with unfavorable eating behaviors, well-being characteristics and weight status, while most of the associations in the case of flexible CR were non-significant. Although flexible CR appears to be a more adaptive weight maintenance strategy compared with rigid control, it does not demonstrate a favorable impact on weight status nor eating behavior and well-being characteristics. Therefore, this calls into question the utility of promoting flexible CR as a public health prevention strategy which might inadvertently promote rigid control as well [22].

### 4.6. Strengths and Limitations

The present validation study has several strengths and limitations. First, it included a general-population-derived sample of both men and women of diverse age and education backgrounds. Moreover, we used a random subsample of the NutriNet-Santé cohort, selected for the purpose of being representative of the French population in terms of age, gender, and level of education [66]. However, from the original, randomly drawn sample of 1000 participants selected for this validation analysis, 38% did not return all questionnaires, therefore the final sample (N = 620) may not be fully representative. Specifically, in the final sample, the distribution of gender and age remained close to that observed in the general French population, while the distribution of educational level diverged noticeably, mainly due to a lower response rate by participants with low formal education. In addition, caution is needed when generalizing our results since the NutriNet-Santé study is a long-term Web-based cohort with participants recruited on a voluntary basis. This implies that the study sample likely represents health-conscious individuals interested in nutritional issues. Next, CFAs were performed using the mean- and variance-weight least-square estimator (WLSMV), which uses the tetrachoric correlation matrix and is better suited for categorical variables compared with the maximum likelihood estimator, which could bias model-data fit [52]. During the translation and cultural adaptation phase, we strove for an equivalence of content between the original and the adapted versions. However, the validation study revealed some challenges regarding the comprehension of several questions which could be due to cultural differences. Some limitations in the study design should also be mentioned. Multiple-group analyses of gender, diet status, or weight status to investigate measurement invariance were not performed because the baseline model presented null frequencies with respect to the cross-classification of certain items. Hypothesis testing analyses were performed using bivariate correlations only, which might have led to biased estimates. Another limit pertains to differences in assessment period between the different psychological traits. Finally, the self-reported anthropometric measures might have led to some misclassification. However, standardized clinical measurements in a subsample (*n* = 2513) of the NutriNet-Santé cohort showed convergence with self-reported data [51].

## 5. Conclusions

In the present study, we translated, adapted, and validated the flexible and rigid CR scales, originally developed by Westenhoefer and colleagues, in a French population-based sample. This 27-item instrument demonstrated good psychometric properties, confirming that flexible and rigid CR are independent constructs. Our data suggested that rigid CR was associated with more underweight or overweight/obesity. In addition, both rigid and flexible CR were associated with rather unfavorable eating behavior and well-being characteristics. The distinct flexible and rigid CR scales may prove useful tools to better understand the role of CR in weight control in the general population as well as in clinical samples. Longitudinal population-based studies, taking into account pertinent confounders, are needed to determine if causal relationships exist between flexible and rigid CR, dietary intake, weight status and eating disorders, and what the direction of causality might be.

## Figures and Tables

**Table 1:** Model fit statistics and reliability estimates obtained from confirmatory factor analyses of the flexible and rigid cognitive restraint scales in two random samples of the NutriNet-Santé study (2017) and comparison with the respective estimates in the original publication.

	Original Model (Westenhoefer et al.) [17]	Respecified Model Subsample 1	Respecified Model Subsample 2
	N = 620	N = 319	N = 301
*χ* ^2^	1237.4	591.4	584.7
df	349	322	322
*χ*^2^/df	3.55	1.84	1.82
CFI	0.93	0.96	0.96
TLI	0.92	0.96	0.95
RMSEA	0.064 [0.060, 0.068]	0.051 [0.045, 0.058]	0.052 [0.045, 0.059]

**Table 2:** Confirmatory factor analysis of the flexible and rigid cognitive restraint (CR) scales among 301 participants of the NutriNet-Santé study (2017).

Item #	Item Label	R ^1^	σ², ²
Flexible Cognitive restraint scale			
FC1	When I have eaten my quota of calories, I am usually good about not eating any more.	0.09	0.99
FC2	I deliberately take small helpings as a means of weight control.	0.72	0.49
FC3	While on a diet, if I eat food that is not allowed, I consciously eat less for a period of time to make up for it.	0.78	0.39
FC4	I consciously hold back at meals in order not to gain weight.	0.73	0.47
FC5	I pay a great deal of attention to changes in my figure.	0.62	0.61
FC6	How conscious are you of what you are eating?	0.31	0.90
FC7	How likely are you to consciously eat less than you want?	0.54	0.71
FC8	If I eat a little bit more on one day, I make up for it the next day.	0.70	0.78
FC9	I pay attention to my figure, but I still enjoy a variety of foods.	0.90	0.62
FC10	I prefer low calorie foods that are not fattening.	0.88	0.39
FC11	If I eat a little bit more during one meal, I make up for it at the next meal.	0.67	0.55
FC12	Do you deliberately restrict your intake during meals even though you would like to eat more?	0.80	0.35
Rigid Cognitive restraint scale			
RC1	I have a pretty good idea of the number of calories in common food.	0.40	0.84
RC2	I count calories as a conscious means of controlling my weight.	0.62	0.62
RC3	How often are you dieting in a conscious effort to control your weight?	0.84	0.29
RC4	Would a weight fluctuation of 5 lb affect the way you live your life?	0.49	0.76
RC5	Do feelings of guilt about overeating help you to control your food intake?	0.55	0.69
RC6	How frequently do you avoid “stocking up” on tempting foods?	0.44	0.81
RC7	How likely are you to shop for low calorie foods?	0.69	0.52
RC8	I eat diet foods, even if they do not taste very good.	0.62	0.61
RC9	A diet would be too boring a way for me to lose weight.	0.19	0.96
RC11	I alternate between times when I diet strictly and times when I don’t pay much attention to what and how much I eat.	0.58	0.66
RC12	Sometimes I skip meals to avoid gaining weight.	0.42	0.83
RC13	I avoid some foods on principle even though I like them.	0.78	0.34
RC14	I try to stick to a plan when I lose weight.	0.74	0.45
RC15	Without a diet I wouldn’t know how to control my weight.	0.47	0.78
RC16	Quick success is most important for me during a diet.	0.91	0.82

^1^ Correlation with the corresponding scale score: rigid scale for RC items, and flexible scale for FC items. ^2^ Variance of each item.

**Table 3:** Differences between flexible and rigid cognitive restraint (CR) dimensions according to socio-demographic and anthropometric characteristics among 620 participants of the NutriNet-Santé study (2017).

	N	Flexible CR (Range: 0–12) ^1^ Mean (SD)	*p*	Rigid CR (Range: 0–15) ^1^ Mean (SD)	*p*
All		5.45 (2.87)		3.41 (2.76)	
Gender			<0.001		<0.001
Male	296	4.76 (2.60)		2.78 (2.40)	
Female	324	6.07 (2.96)		4.00 (2.93)	
Age			0.004		0.42
<31y	70	5.03 (2.61)		3.93 (3.20)	
31–50y	194	4.97 (2.72)		3.22 (2.72)	
51–65y	188	5.74 (2.94)		3.44 (2.59)	
>65y	168	5.92 (2.9)		3.40 (2.78)	
Educational level (%)			0.25		0.87
Primary	43	5.19 (2.82)		3.37 (2.80)	
Secondary	349	5.34 (2.91)		3.34 (2.72)	
Undergraduate	106	5.96 (2.90)		3.55 (2.78)	
Postgraduate	122	5.38 (2.72)		3.53 (2.85)	
Occupational status (%)			0.084		0.11
Unemployed	73	5.21 (3.18)		3.62 (3.14)	
Student	14	5.86 (3.37)		5.93 (4.71)	
Self-employed, farmer	20	5.15 (2.60)		2.50 (1.99)	
Employee, manual worker	149	5.14 (2.85)		3.44 (2.73)	
Intermediate professions	65	5.06 (2.28)		2.95 (2.27)	
Managerial staff, intellectual profession	77	5.12 (2.71)		3.23 (2.43)	
Retired	221	5.95 (2.94)		3.46 (2.71)	
Body mass index (%)			0.10		0.001
Underweight (<18.5 kg/m^2^)	27	5.89 (3.21)		3.89 (4.14)	
Normal (≥18.5 and <25 kg/m^2^)	333	5.37 (2.73)		3.01 (2.53)	
Overweight (≥25 and <30 kg/m^2^)	177	5.79 (2.98)		3.73 (2.75)	
Obese (≥30 kg/m^2^)	83	4.86 (2.97)		4.20 (2.85)	
History of dieting to lose weight			0.013		<0.001
Never dieter	516	5.32 (2.86)		3.20 (2.59)	
Dieter	104	6.09 (2.84)		4.48 (3.27)	

^1^ Higher scores indicate greater flexible or rigid CR.

**Table 4:** Pearson’s correlation coefficients between the flexible and rigid cognitive restraint (CR) scales and different eating behaviors and psychological well-being characteristics, such as cognitive restraint, uncontrolled eating, emotional eating, intuitive eating, eating disorders, self-esteem, impulsivity, satisfaction with life and optimism, in the NutriNet-Santé study (2017).

		Flexible CR		Rigid CR	
	N	r	*p*	r	*p*
Three-Factor Eating Questionnaire (TFEQ-R21)					
Cognitive restraint	551	0.53	<0.001	0.52	<0.001
Emotional eating	551	0.09	0.038	0.26	<0.001
Uncontrolled eating	551	−0.05	0.21	0.14	<0.001
Intuitive eating (IES-2)	522	−0.21	<0.001	−0.43	<0.001
Eating for physical rather than emotional reasons	522	−0.09	0.043	−0.28	<0.001
Reliance on hunger and satiety cues	522	−0.05	0.31	−0.21	<0.001
Unconditional permission to eat	522	−0.45	<0.001	−0.50	<0.001
Impulsivity (BIS-11)	534	−0.10	0.025	0.05	0.25
Eating disorders (SCOFF)	547	0.18	<0.001	0.43	<0.001
Self-esteem (RSES)	520	−0.02	0.65	−0.12	0.005
Satisfaction with life (SWLS)	520	0.03	0.46	−0.15	<0.001
Optimism (LOT-R)	520	−0.02	0.65	−0.15	<0.001

## Data Availability

If you are a researcher of a public institution, you can submit a collaboration request including your institution and a brief description of your project to collaboration@etude-nutrinet-sante. All requests will be reviewed by the steering committee of the NutriNet-Santé study. A financial contribution may be requested. If the collaboration is accepted, a data access agreement will be necessary and appropriate authorizations from the competent administrative authorities may be needed. In accordance with existing regulations, no personal data will be accessible.

## Supplementary Materials

The following supporting information can be downloaded at: https://www.mdpi.com/article/10.3390/ijerph191912519/s1, Table S1: French version of the flexible and rigid cognitive restraint scale; Table S2: English version of the flexible and rigid cognitive restraint scale. References [17,33] are cited in the supplementary materials.

## Abbreviations

CR: cognitive restraint; TFEQ, Three-Factor Eating Questionnaire; IRB, Institutional Review Board of the French Institute for Health and Medical Research; CNIL, Commission Nationale Informatique et Libertés; BMI, Body Mass Index.
