# Supplementary material for: Validation of the Flexible and Rigid Cognitive Restraint Scales in a General French Population
Source: Int J Environ Res Public Health. 2022 Sep 30;19(19):12519. doi: 10.3390/ijerph191912519 (PMC9564632; doi:10.3390/ijerph191912519)
Supplement: Supplementary file 1 [file ijerph-19-12519-s001.zip › ijerph-1843575-supplementary.pdf]

SUPPLEMENTAL TABLE S1

French version of the flexible and rigid cognitive restraint scale

Item #	Item
Flexible Cognitive restraint scale	
FC1	Une fois que j'ai mangé la quantité de calories dont j'ai besoin, je parviens habituellement facilement à ne plus manger. *(vrai, faux)*
FC2	À table, je prends délibérément de petites parts comme moyen de contrôler mon poids. *(entièrement vrai, assez vrai, assez faux, complètement faux)*
FC3	Lorsque je suis au régime, si je mange quelque chose qui ne m'est pas autorisé, je mange volontairement moins pendant un certain temps pour me rattraper. *(vrai, faux, je n'ai jamais fait de régime)*
FC4	À table, je me retiens volontairement de manger pour ne pas prendre de poids. *(entièrement vrai, assez vrai, assez faux, complètement faux)*
FC5	Je fais très attention aux changements de ma silhouette. *(vrai, faux)*
FC6	A quel point êtes-vous conscient.e de ce que vous mangez? *(pas du tout, un peu, assez, beaucoup)*
FC7	Avez-vous tendance à manger volontairement moins que vous n'en avez envie ? *(pas du tout, un peu, modérément, fortement)*
FC8	Si je mange un peu plus un jour, je compense le lendemain. *(vrai, faux)*
FC9	Je fais attention à ma ligne, mais je mange quand même varié. *(vrai, faux)*
FC10	Je préfère manger des aliments peu caloriques qui ne font pas grossir. *(vrai, faux)*
FC11	Si je mange un peu plus pendant un repas, je compense au repas d'après. *(vrai, faux)*
FC12	Est-ce que vous limitez délibérément ce que vous mangez pendant les repas, même si vous aimeriez manger davantage ? *(jamais, parfois, souvent, toujours)*
Rigid Cognitive restraint scale	
RC1	J'ai une assez bonne idée du nombre de calories contenues dans les aliments courants *(vrai, faux)*
RC2	Je compte délibérément les calories pour contrôler mon poids. *(vrai, faux)*
RC3	A quelle fréquence faites-vous délibérément un régime pour contrôler votre poids ? *(jamais ou rarement, parfois, assez souvent, tout le temps ou presque)*
RC4	Une variation de poids de 2,5 kg modifierait-elle votre façon de vivre ? *(pas du tout, un peu, assez, beaucoup)*
RC5	Le fait que vous vous sentiez coupable de trop manger vous aide-t-il à contrôler votre alimentation? *(pas du tout, un peu, assez, beaucoup, je ne me sens jamais coupable)*
RC6	Vous arrive-t-il d'éviter de « faire des provisions » d'aliments qui vous tentent ? *(jamais ou presque, rarement, souvent, presque toujours)*
RC7	Quand vous faites les courses, avez-vous tendance à acheter des aliments "basses-calories"? *(jamais ou rarement, parfois, assez souvent, tout le temps ou presque)*
RC8	Je mange des aliments allégés, même s'ils n'ont pas très bon goût. *(vrai, faux)*
RC9	Un régime serait un moyen trop ennuyeux pour moi de perdre du poids. *(vrai, faux)*
RC11	J'alterne entre des périodes où je suis au régime de façon stricte et des périodes où je ne fais pas vraiment attention à ce que je mange ni à la quantité. *(vrai, faux)*
RC12	Je saute parfois des repas pour éviter de prendre du poids.

	(vrai, faux)
RC13	J'évite certains aliments par principe même si je les aime.<br>*(vrai, faux)*
RC14	J'essaie de me tenir à un programme quand je veux perdre du poids.<br>*(vrai, faux, je n'ai jamais essayé de perdre du poids)*
RC15	Sans faire de régime, je ne saurais pas comment contrôler mon poids.<br>*(vrai, faux)*
RC16	Le plus important pour moi quand je fais un régime est de réussir vite.<br>*(vrai, faux, je n'ai jamais fait de régime)*

All items and response modalities are French translations of items by Westenhoefer (17), except for FC2 and FC4 which are from the TFEQ-R21 (33). A few changes in item formulations, in accordance with French language use conventions, were performed, as follows: "my quota of calories" was changed to "the quantity of calories I need" (FC1), "light food" was changed to "low-calorie food" (FC10), "without a diet plan" was changed to "without a diet" (RC15). Some response modalities were slightly modified: a third modality was added for individuals who never feel guilty (RC5), never attempted to lose weight (RC13) or who were never on a diet (FC3, RC15); response modalities for FC12 were reversed; response modalities for FC6, RC3, RC4, RC6, RC7 were slightly changed in order to cover a wider range of behaviors. All items are dichotomously coded for the analysis. Underlined modalities are scored 1. The total score is obtained by adding up all individual item ratings and then dividing the result by the number of items in each scale, for a final score ranging from 0 to 8 for the flexible CR, and from 0 to 9 for the rigid CR (higher scores indicating greater CR).

SUPPLEMENTAL TABLE S2

English version of the flexible and rigid cognitive restraint scale

Item #	Item
Flexible Cognitive restraint scale	
FC1	When I have eaten my quota of calories, I am usually good about not eating any more.<br>*(true, false)*
FC2	I deliberately take small helpings as a means of weight control.<br>*(definitely true, mostly true, mostly false, definitely false)*
FC3	While on a diet, if I eat food that is not allowed, I consciously eat less for a period of time to make up for it.<br>*(true, false, I have never followed a diet)*
FC4	I consciously hold back at meals in order not to gain weight.<br>*(definitely true, mostly true, mostly false, definitely false)*
FC5	I pay a great deal of attention to changes in my figure.<br>*(true, false)*
FC6	How conscious are you of what you are eating?<br>*(not at all, slightly, moderately, very much)*
FC7	How likely are you to consciously eat less than you want?<br>*(unlikely, slightly unlikely, moderately likely, very likely)*
FC8	If I eat a little bit more on one day, I make up for it the next day.<br>*(true, false)*
FC9	I pay attention to my figure, but I still enjoy a variety of foods.<br>*(true, false)*
FC10	I prefer low calorie foods that are not fattening.<br>*(true, false)*
FC11	If I eat a little bit more during one meal, I make up for it at the next meal.<br>(true – false)
FC12	Do you deliberately restrict your intake during meals even though you would like to eat more?<br>*(never, rarely, often, always)*
Rigid Cognitive restraint scale	
RC1	I have a pretty good idea of the number of calories in common food.<br>*(true, false)*
RC2	I count calories as a conscious means of controlling my weight.<br>*(true, false)*
RC3	How often are you dieting in a conscious effort to control your weight?<br>*(never or rarely, sometimes, usually, almost always)*
RC4	Would a weight fluctuation of 5 lb affect the way you live your life?<br>*(not at all, slightly, moderately, very much)*
RC5	Do feelings of guilt about overeating help you to control your food intake?<br>*(not at all, slightly, moderately, very much, I never feel guilty)*
RC6	How frequently do you avoid “stocking up” on tempting foods?<br>*(never or almost never, seldom, usually, almost always)*
RC7	How likely are you to shop for low calorie foods?<br>*(never or almost never, seldom, usually, always or almost always)*
RC8	I eat diet foods, even if they do not taste very good.<br>*(true, false)*
RC9	A diet would be too boring a way for me to lose weight.<br>*(true, false)*
RC11	I alternate between times when I diet strictly and times when I don’t pay much attention to what and how much I eat.<br>*(true, false)*
RC12	Sometimes I skip meals to avoid gaining weight.<br>*(true, false)*

RC13	I avoid some foods on principle even though I like them.<br>*(true, false)*
RC14	I try to stick to a plan when I lose weight.<br>*(true, false, I never attempted to lose weight)*
RC15	Without a diet I wouldn't know how to control my weight.<br>*(true, false)*
RC16	Quick success is most important for me during a diet.<br>*(true, false, I have never followed a diet)*

All items and response modalities are French translations of items by Westenhoefer (17), except for FC2 and FC4 which are from the TFEQ-R21 (33). A few changes in item formulations, in accordance with French language use conventions, were performed, as follows: "my quota of calories" was changed to "the quantity of calories I need" (FC1), "light food" was changed to "low-calorie food" (FC10), "without a diet plan" was changed to "without a diet" (RC15). Some response modalities were slightly modified: a third modality was added for individuals who never feel guilty (RC5), never attempted to lose weight (RC13) or who were never on a diet (FC3, RC15); response modalities for FC12 were reversed; response modalities for FC6, RC3, RC4, RC6, RC7 were slightly changed in order to cover a wider range of behaviors. All items are dichotomously coded for the analysis. Underlined modalities are scored 1. The total score is obtained by adding up all individual item ratings and then dividing the result by the number of items in each scale, for a final score ranging from 0 to 8 for the flexible CR, and from 0 to 9 for the rigid CR (higher scores indicating greater CR).
